# Mitochondrial Dynamics and mRNA Translation: A Local Synaptic Tale

**DOI:** 10.3390/biology13090746

**Published:** 2024-09-23

**Authors:** Marta Zaninello, Pedro Baptista, Filipe V. Duarte

**Affiliations:** 1 Institute for Genetics, University of Cologne, 50931 Cologne, Germany; mzaninel@uni-koeln.de; 2 Cologne Excellence Cluster on Cellular Stress Responses in Aging-Associated Diseases (CECAD), 50931 Cologne, Germany; 3 CNC—Center for Neuroscience and Cell Biology, University of Coimbra, 3004-504 Coimbra, Portugal; junior.fonseca2002@gmail.com; 4 Department of Life Sciences, University of Coimbra, 3004-504 Coimbra, Portugal

**Keywords:** mitochondria, mitochondrial morphology, mRNA, mRNA trafficking, neuron, synapse

## Abstract

**Simple Summary:**

Mitochondria-shaping proteins are pivotal in maintaining the dynamic structure of mitochondria, which constantly undergo fusion and fission. This dynamic reshaping is crucial for adapting to the energetic demands of neurons, especially in the axons and dendrites, where mitochondria play vital roles in synaptic activity. Therefore, the precise localization and timely translation of mitochondria-shaping proteins are essential for neuronal resilience and the overall health of the nervous system. Transport granules, which are membrane-less organelles formed through liquid–liquid phase separation, are responsible for transporting these mRNAs. These granules contain RNA-binding proteins (RBPs) that stabilize and regulate mRNA content, ensuring the proper translation of mitochondrial proteins at specific sites. Another mechanism involves tethering mRNAs to organelles such as mitochondria, endosomes, and lysosomes, allowing for rapid and efficient transport in response to cellular signals. Disruptions in these processes can lead to impaired mitochondrial dynamics, contributing to neuronal dysfunction and neurodegenerative diseases such as Alzheimer’s and Parkinson’s.

**Abstract:**

Mitochondria are dynamic organelles that can adjust and respond to different stimuli within a cell. This plastic ability allows them to effectively coordinate several cellular functions in cells and becomes particularly relevant in highly complex cells such as neurons. An imbalance in mitochondrial dynamics can disrupt mitochondrial function, leading to abnormal cellular function and ultimately to a range of diseases, including neurodegenerative disorders. Regulation of mRNA transport and local translation inside neurons is crucial for maintaining the proteome of distal mitochondria, which is vital for energy production and synaptic function. A significant portion of the axonal transcriptome is dedicated to mRNAs for mitochondrial proteins, emphasizing the importance of local translation in sustaining mitochondrial function in areas far from the cell body. In neurons, local translation and the regulation of mRNAs encoding mitochondrial-shaping proteins could be essential for synaptic plasticity and neuronal health. The dynamics of these mRNAs, including their transport and local translation, may influence the morphology and function of mitochondria, thereby affecting the overall energy status and responsiveness of synapses. Comprehending the mitochondria-related mRNA regulation and local translation, as well as its influence on mitochondrial morphology near the synapses will help to better understand neuronal physiology and neurological diseases where mitochondrial dysfunction and impaired synaptic plasticity play a central role.

## 1. Intro: The Role of Mitochondria in Neurons

The brain’s energy consumption is quite high, accounting for approximately 20% of the body’s total metabolic energy despite comprising only approximately 2% of its total mass. Neurons are responsible for most of this energy use, primarily to restore ion gradients across the plasma membrane, which are altered by the creation of action potentials and synaptic transmission [1]. Neurons contain numerous mitochondria, which play a crucial role in maintaining neuronal integrity and function under normal conditions, particularly at the synapse [2], namely serving a vital function in energy buffering [3,4].

**Figure 1:**
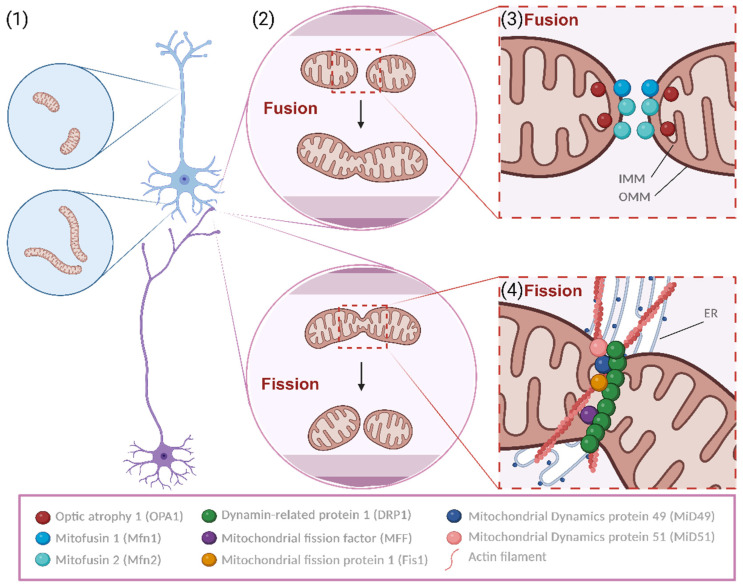
Mitochondrial fusion and fission. Mitochondria display distinct morphologies and distributions in axons and dendrites (1). Generally, dendritic mitochondria display long and tubular shapes, forming a complex network within the dendritic arbor, while axonal mitochondria display a remarkably small sand punctate morphology, occupying a small fraction of the axonal volume. Moreover, mitochondria are dynamic organelles that change morphology (2). They undergo cyclic processes of specialized structural alterations, namely fusion and fission, which depend on particular proteins, pro-fusion or pro-fission. Fusion is dependent on the tight control and activity of Mitofusins and OPA1, leading to the fusing of MIM and MOM, respectively (3). Regarding fission, a synchronous regulation of actin nucleation aided by the ER and the recruitment of DRP1 for the MOM via the variously described adaptors is needed to rupture mitochondria (4). This cyclic process is crucial for the well-being of mitochondria and, hence, also for the physiologic functioning of neurons. Created using BioRender.com.

Mitochondria exhibit unique shapes and distributions in axons versus dendrites. In pyramidal neurons, the primary excitatory type in the cerebral cortex, dendritic mitochondria are long and tubular, forming a network that fills 70–80% of the dendritic arbor and often lies near spines [5]. Conversely, axonal mitochondria are small, punctate, and occupy less than 10% of the axonal volume. Mitochondrial morphology in neurons, akin to other cell types, is regulated by fusion and fission mechanisms, collectively known as mitochondrial dynamics [6] (Figure 1). This suggests that the morphological differences between axons and dendrites stem from differential regulation of mitochondrial dynamics. Notably, presynaptic mitochondrial size and axon branching are regulated by fission in cortical pyramidal neurons [6,7].

**Figure 2:**
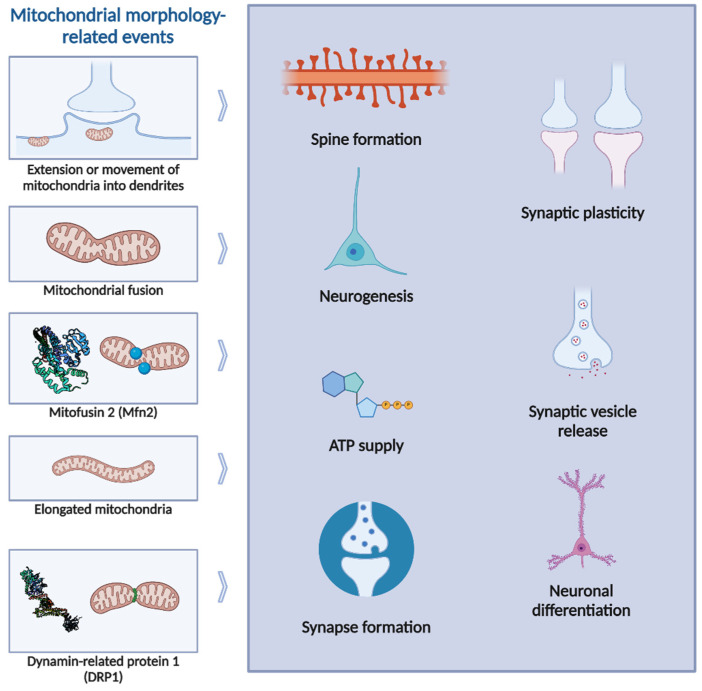
Mitochondrial morphology-related events and neuronal regulation. Mitochondrial fusion has been widely implicated in several processes, such as neuronal differentiation, dendritic arborization, or spine formation. Particularly, Mfn2-dependent mitochondrial fusion has been implicated in neuronal modulation, as well as in disease: decreased protein expression of Mfn1, Mfn2 and OPA1 has been reported in Alzheimer’s disease (AD), Huntington’s disease (HD) and autosomal dominant optic a Atrophy (reviewed in [62]).Mitochondrial fission, and particularly DRP1-dependent fission, has also been reported to regulate neuronal function and synaptic activity. Alzheimer’s disease can affect mitochondrial fission: oligomeric Aβ increases DRP1 through nitric oxide (NO) signaling pathways [68], while subsequent tau pathology reduces DRP1 activity by enhancing its propensity to remain bound to actin, further contributing to neuronal degeneration [69]. In AD and HD, aberrant mitochondrial dynamics have also been observed, characterized by elevated protein levels of DRP1 and Fis1 [62]. Created using BioRender.com.

Alterations in mitochondrial morphology and function affect neuronal physiology and synaptic transmission, positioning mitochondria as neuromodulators [4] (Figure 2). Specifically at the presynaptic terminal, mitochondria are essential for neurotransmitter release by supplying ATP and buffering local calcium (Ca^2+^) content [8]. Moreover, synaptic vesicle turnover and release are significantly affected when mitochondrial function is inhibited, and ATP is depleted [9]. The density and rate of mitochondrial oxidative phosphorylation can affect presynaptic ATP regeneration and, consequently, synaptic vesicle exocytosis [9]. However, a recent study reported that the majority (~80–90%) of axonal mitochondria in cortical pyramidal neurons (CPNs) lack mitochondrial DNA (mtDNA); moreover, the authors demonstrated that in axons of CPNs but not in their dendrites mitochondrial complex V (ATP synthase) functions in a reverse way, consuming ATP [10].

In the post-synaptic compartment, a recent study in *C. elegans* has shown that mitochondrial ROS signaling regulates excitatory synapse function by altering the recruitment of glutamate receptors, thereby integrating several mitochondrial functions from energy production to Ca^2+^ buffering and ROS signaling [11].

Electron microscopy has significantly advanced our understanding of mitochondria’s anatomy and ultrastructure [12]. It is now well-established that mitochondrial structure closely correlates with its functional state. Studies comparing mitochondria ultrastructure revealed that presynaptic mitochondria in highly active neurons are larger and contain more densely packed lamellar cristae than those in less active neurons [13] and that presynaptic mitochondria are generally smaller and darker compared with post-synaptic mitochondria [14]. Due to the limitations of 2D snapshots in capturing the complex morphology of axons and dendrites, 3D reconstructions of mitochondrial networks in the mouse brain have been conducted. These studies found that mitochondrial morphology varies across subcellular compartments. Specifically, presynaptic mitochondria are generally smaller, shorter, and confined to presynaptic terminals, while most post-synaptic mitochondria are larger and extend through significant portions of the dendrites [15,16]. Additionally, age-related morphological changes affect the status of the neuronal mitochondrial network [17].

Synaptic mitochondria are primarily generated in the neuron’s cell body and transported to axons or dendrites via motor proteins on microtubule tracks, with synapse formation significantly boosting their bidirectional movement [18]. These mitochondria might have a longer lifespan and experience more oxidative damage compared with those in neuronal soma or glial cells. Although the unique bioenergetics and diversity of synaptic mitochondria are poorly understood, certain disparities with non-synaptic mitochondria have been identified [19,20,21]. Recent findings show that mitochondrial long-lived proteins (LLPs) in long-lived cell tissues like the brain can last for months [22]. Despite mixed findings on mRNA transport and mitochondrial protein translation in neurons, specific durable mitochondrial proteins are crucial for maintaining mitochondrial structure in neurons. Graham et al. discovered distinct proteomic profiles for synaptic versus non-synaptic mitochondria, noting that modifying intrinsic mitochondrial proteins can alter synaptic morphology in vivo [23]. Furthermore, an investigation revealed an uneven distribution of synapses with different protein lifespans across neurons and brain areas [24]. The exact mechanisms differentiating synaptic from non-synaptic mitochondria are not fully understood, yet their Ca^2+^ buffering capacity appears central [25,26].

In this review, we discuss the latest findings regarding mitochondrial dynamics-related mRNA regulation and protein translation in the synapse.

## 2. Mitochondrial Dynamics in the Synapse

Mitochondria are dynamic organelles that change morphology and intracellular distribution by responding to heterogeneous external cues associated with varied degrees of cellular energetic needs [27]. They undergo cyclic processes of specialized structural alterations in response to external cues, namely fission and fusion, interacting with each other and forming an innate dynamic process that needs constant equilibrium to maintain the physiological function of the cell [28]. The fusion and fission mechanisms depend on the precise regulation of molecular markers, with mechanisms conserved in different cell types but having a specific role in dendritic spines.

### 2.1. Regulators of Mitochondrial Fusion and Fission

Mitochondria have a double-membrane structure, encompassing an outer mitochondrial membrane (MOM) and an inner mitochondrial membrane (MIM), divided by an intermembrane space (IMS) (Figure 1).

Mitochondrial fusion is a mechanism described as the close contact tethering and merging of two mitochondria and the posterior emergence of a unitary larger organelle from the regulated fusion of the membrane of mitochondria. Two major proteins are responsible for the processing of said mechanism: Mitofusin 1 and 2 (Mfn1 and Mfn2) are large GTPases responsible for the fusing of MOMs from different mitochondria, stabilizing the interaction of the mitochondria in contact by enabling the interaction and rearrangement of MIMs [29]. Then, optic atrophy type 1 protein (OPA1), a mechano-chemical GTPase, physically adapts the inner membrane, granting a functional restructuring of this lipidic structure with the contacted mitochondrion whilst maintaining the protein content and functionality of this relevant membrane of mitochondrial innate capacity to metabolically support cellular function [30]. Moreover, OPA1 promotes the reshaping of the internal mitochondrial cristae, enabling the maintenance of its function [31]. Fusion is important for synthesizing ATP because the fusion of healthy mitochondria leads to an increase in the cristae area, imposing considerably higher pumping of H^+^, which is processed via ATPases in higher amounts, thereby increasing energy production. In this way, mitochondria support demanding cellular processes such as apoptosis and mitotic progression [32].

Fission is a mitochondrial mechanism that counteracts fusion. It is described as the separation of mitochondrial membranes, mediated by specific molecular players. It is a heterogeneous process within species, but in humans, it is dependent on the endoplasmic reticulum (ER). The ER tags the target mitochondria by interacting with membrane lipids and stimulating the formation of actin bands near the fission site. During this actin rings’ assembly in a putative context, dynamin-related protein 1 (DRP1) is activated by phosphorylation in the residue Ser616 (phosphorylation that renders the protein more active) and can interact with mitochondrial receptors present at the MOM, such as mitochondrial fission factor (MFF) [33,34]. This interaction is also mediated by regulators such as mitochondrial fission 1 (Fis1), mitochondrial dynamics protein 49 and 51 (MiD 49 and MiD51), and MFF itself, receptors of DRP1 [35]. This process of actin band strangling, with the addition of DRP1 GTPase activity on MOM regulated by MFF, leads to the constraining and eventual division of the mitochondrial membrane, forming separate organelles. Fission is thought to be highly important in the synaptic context as it might facilitate mitochondrial transport, boosting the production of energy in confined spaces with decreased accessibility [4]. Besides that, fission is also extremely important for mitochondrial division, enabling the formation of new mitochondria [36].

### 2.2. Role of Mitochondrial Morphology in the Synapse

Mitochondria have distinct and equally relevant roles, namely oxidative, metabolic, and Ca^2+^ buffering functions. Therefore, it’s easily understood that mitochondria operate to regulate and modify synaptic transmission and also related processes of functional and structural plasticity [9,26,37,38,39].

A connection between alterations in mitochondrial function and synaptic activity has been reported long ago for peripheral synapses [40]. Also, some pharmacological studies have shown that inhibiting mitochondrial activity impairs synaptic potentiation and neurotransmission [41,42]. Additionally, acutely blocking mitochondrial function during intense stimulation leads to depressed synaptic transmission [43]. Interestingly, a couple of decades ago, Li et al. reported that enhancing mitochondrial respiration can increase the number and plasticity of spines and synapses [44]. The extension or movement of mitochondria into dendritic protrusions correlates with the development and morphological plasticity of spines, whereas molecular manipulations of DRP1 and OPA1 proteins, reducing dendritic mitochondria content, lead to loss of synapses and dendritic spines. Thus, dendritic mitochondria have been found to be crucial for the support of synapses. Reciprocally, synaptic activity modulates the motility and fusion/fission balance of mitochondria and controls mitochondrial distribution in the dendrites. These findings indicate that mitochondria not only respond to synaptic activity in various ways but are also able to regulate synaptic plasticity [45]. Indeed, a recent study from Thomas et al. has shown that post-synaptic mitochondria are critical for the development, plasticity, and maintenance of synaptic inputs in cortical neurons [46]. This study reported that synapses are larger and exhibit greater selectivity to visual stimuli in spines with a mitochondrion in the head or neck, suggesting that mitochondria support the structurally and functionally diverse inputs innervating the basal dendrites. Mitochondrial fusion has already been shown to be necessary for synapse formation in human-induced pluripotent stem cells-derived cortical neurons [47]. Mfn2 has been demonstrated to be essential for human mitochondrial development in neuronal maturation and differentiation, and Mfn2 knockdown impacts neurogenesis and synapse formation. More recently, Kochan et al. reported that mitochondrial fusion promotes elongated mitochondria in dendrites of new neurons and that Mfn1 or Mfn2 absence abrogates synaptic plasticity in hippocampal neurons [48]. Moreover, it has been recently shown in vivo that the local regulation of fusion-fission balance in the hippocampus is responsible for the distinct mitochondrial morphology in dendrites. Interestingly, Virga et al. have shown that mitochondria within the dendritic arbor display a further degree of subcellular, layer-specific distinct morphology in the hippocampus [49]. In the dendrites of these neurons, mitochondria morphology ranges from highly fused and elongated in the apical tuft to more fragmented in the apical oblique and basal dendritic compartments. Additionally, the authors demonstrated that the regulation of this compartment-specific mitochondrial morphology in dendrites requires the activation of AMPK and its ability to phosphorylate two direct effectors: MFF and the anti-fusion, OPA1-inhibiting protein, mitochondrial fission regulator 1-like protein (Mtfr1l).

While the significance of mitochondrial dynamics is well-documented, the importance of mitochondrial fission is less well-defined compared with that of mitochondrial fusion (Figure 2). Primary cultures of neural cells derived from mice lacking DRP1 exhibited a reduced number of neurites and defective synapse formation, emphasizing the crucial role of DRP1-mediated mitochondrial fission in highly polarized cells such as neurons [50]. Moreover, the loss of DRP1 also impairs synaptic vesicle release [51]. This further supports the idea that presynaptic mitochondria play a key role in exocytosis. In accordance, it has also been reported that the ATP supply provided by presynaptic mitochondria is essential for fueling the assembly of the actin cytoskeleton [52] and that the concentration of ATP influences the grouping of synaptic vesicles and mitochondria at synapses [53], as well as the effective movement of synaptic vesicles into the readily releasable pool [54]. Mitochondria provide a novel mechanism for regulating exocytosis by taking up Ca^2+^ from the cytoplasm in response to neuronal activity. This activity leads to substantial increases in presynaptic intracellular Ca^2+^ concentrations, which locally promote synaptic vesicle exocytosis and affect various signaling pathways. One primary mechanism involves the activation of synaptotagmins, which are Ca^2+^ sensors that facilitate the membrane fusion machinery necessary for neurotransmitter release [55]. Additionally, presynaptic Ca^2+^ dynamics are crucial in asynchronous neurotransmitter release, which can last for seconds following repetitive stimulation [56]. Furthermore, presynaptic receptors can regulate Ca^2+^ channel activity, thereby influencing Ca^2+^ influx and neurotransmitter release. This regulation often involves G protein-mediated mechanisms that alter the voltage dependence of Ca^2+^ channels and can also affect K^+^ channels, providing a synergistic mechanism for controlling Ca^2+^ influx and neurotransmitter release [57].

Mitochondria govern these processes by regulating [Ca^2+^]_i_ levels through their negative membrane potential, which is generated by the electron transport chain (ETC), plasma membrane Ca^2+^-ATPases, and the endoplasmic reticulum [58]. However, not all presynaptic terminals contain mitochondria, despite their growing recognition as crucial for maintaining neuronal homeostasis (reviewed in [59]). Indeed, research has shown that preserving a pool of presynaptic mitochondria enhances the stability of presynaptic strength by sustaining local ATP homeostasis [60]. Post-synaptic mitochondria are also important for local functions, as experimentally disrupting dendritic mitochondria results in the loss of dendritic spines and their synapses [61]. Consequently, it is widely accepted that mitochondrial dysfunction and subsequent synaptic dysfunction contribute to a variety of neurological defects [62].

Studies have shown that KCl-induced depolarization of cultured hippocampal neurons increases the fission-to-fusion ratio and enhances mitochondrial distribution in dendritic spines [44]. Changes in mitochondrial dynamics were also examined during synaptic plasticity induction in cultured hippocampal neurons exposed to a Na^+^-salt solution without Mg^2+^ and supplemented with glycine [63]. This treatment led to a quick increase in dendritic mitochondrial fission and elevated mitochondrial matrix Ca^2+^ levels. The fission process was triggered by increased [Ca^2+^]_i_ and facilitated by CaMKII activation. Additionally, DRP1 and dynamin 2 mediated the induced mitochondrial fission. Importantly, blocking fission prevented spine structural changes, and related studies using hippocampal slices revealed that mitochondrial fission plays a role in high-frequency presynaptic stimulation-induced LTP of CA1 synapses [63]. These findings align with evidence showing impaired spatial working memory in mice with DRP1 deletion in postmitotic adult mouse forebrain neurons [64]. The crucial role of mitochondrial fission in synaptic regulation was further demonstrated in cultured hippocampal neurons expressing a dominant negative form of DRP1 (DRP1-K38A). This construct reduced mitochondrial presence in dendrites and spines, decreased dendritic spine numbers, and inhibited activity-induced increases in post-synaptic puncta observed in immunocytochemistry experiments [44]. Furthermore, studies on mice with postnatal DRP1 deletion showed decreased axonal ATP production, impaired synaptic transmission, and compromised hippocampus-dependent memory [65]. Notably, DRP1 knockdown in D1-medium spiny neurons of the *nucleus accumbens* inhibited cocaine-seeking behavior following drug self-administration [66]. PINK1 is essential for activating DRP1 through Ser616 phosphorylation, which promotes synaptic development and plasticity [67]. This study found that DRP1S616A knock-in mice exhibited impaired contextual fear memory and spatial memory, emphasizing the importance of this DRP1 phosphorylation site in learning and memory processes [67].

## 3. Mitochondrial Morphology: A Role in Disease

Since synapses have high energy demands, and neurons may rely on alternative ways to produce the energy necessary to sustain the presynaptic vesicle cycle, neuronal mitochondria are essential for the maintenance of neurotransmission and synaptic plasticity. Consequently, the dysregulation of neuronal mitochondrial function and also mitochondrial morphology have been implicated in neurodegenerative diseases and other neurological disorders [62].

Neurodegenerative diseases, including Alzheimer’s disease (AD), Parkinson’s disease (PD), Huntington’s disease (HD), and amyotrophic lateral sclerosis (ALS), are characterized by the loss of selective neuron subtypes in the CNS. Mitochondrial dynamics-related proteins, often part of the so-called mitochondrial quality control (MQC) mechanisms, play a significant role in the progression of these diseases. Considering AD as an example, mitochondrial fission and fusion proteins are disturbed in the hippocampus in several AD animal models and patients. Specifically, the fission protein Fis1 is increased, while the fusion proteins Mfn1, Mfn2, and OPA1 are decreased [70]. Additionally, the phosphorylation of DRP1 at Ser616 is higher in the brains of AD patients [70]. Furthermore, it has been shown in vivo that the functional regulation of DRP1 by phosphorylation mediated by PINK1 modulates synaptic development and plasticity, further connecting mitochondrial dynamics and neural circuitry formation and refinement [67]. Another study in vivo found significant mitochondrial fragmentation and irregular distribution of mitochondria in the pyramidal neurons of the brains of mice with AD before the accumulation of amyloid plaques [71]. By using the mitochondrial division inhibitor-1 (Mdivi-1), a pharmacological inhibitor of DRP1, the mitochondrial fragmentation and distribution issues, as well as mitochondrial function, were resolved. Moreover, Mdivi-1 led to a decrease in extracellular amyloid deposits, prevented cognitive decline, and increased cortical levels of synaptophysin [71].

In an animal model of traumatic brain injury (TBI), DRP1-dependent mitochondrial alterations have been associated with intense memory impairments [72]. DRP1 levels in purified hippocampal mitochondria were increased in animals with TBI compared with sham controls. Additionally, cryo-electron micrographs of these mitochondria showed that TBI resulted in an initial increase in the length of hippocampal mitochondria 24 h post-injury, followed by a significant decrease in mitochondrial length 72 h post-injury. Post-TBI administration of Mdivi-1 prevented this decrease in mitochondrial length. Mdivi-1 treatment also reduced the loss of newborn neurons in the hippocampus and improved novel object recognition (NOR) memory and context-specific fear memory [72]. These findings indicate that TBI increases mitochondrial fission and that inhibition of fission improves hippocampal-dependent learning and memory, suggesting that strategies to reduce mitochondrial fission may have translational value after injury.

Ischemic stroke is another major cause of death and disability of the nervous system. Although reperfusion through revascularization has reduced the mortality rate of ischemic stroke, it also leads to additional damage to the brain tissue, known as ischemia-reperfusion (I/R) injury. Several processes related to mitochondrial quality control (MQC) have been suggested to contribute to I/R damage. During cerebral I/R injury, mitochondrial fission and mitophagy help to remove damaged mitochondria and promote mitochondrial apoptosis of neuron cells [73,74]. Overexpression of Sirtuin 3 (Sirt3) can inhibit mitochondrial fission and attenuate cerebral ischemia-reperfusion injury by triggering pro-survival signals in neurons subjected to I/R injury [75].

Epilepsy is characterized by recurrent seizures that affect neurons without provocation. Research suggests that mitochondrial stress and MQC play a role in the development of this condition. Studies have shown that the levels of OPA1, Mfn2, MFF, and DRP1 are elevated in a mouse model of epilepsy [76]. Additionally, the serine peptidase LONP1, which is also related to MQC, is upregulated in the mitochondria during *status epilepticus* (SE), and its knockdown has been shown to increase SE-induced mitochondrial apoptosis in neurons [77].

Mitochondrial quality control-related gene mutations or abnormal expression have also been reported to be involved in several brain dysplasias [78]. Heterozygous or *de novo* mutations in the *dnm1l* gene, coding for DRP1 protein, have been shown to cause microcephaly, optic atrophy, hypoplasia, epileptic encephalopathy, and neurodevelopment delay [79,80,81]. Specifically regarding mitochondrial morphology proteins (also reviewed in [62]), the overexpression of Mfn2 mutation in cultured neurons has been reported to disrupt axonal mitochondrial positioning and to promote axon degeneration [82]. Moreover, in a neuron-specific knockout mouse model, it has been shown that Mfn2 is required for dendritic outgrowth, axonal projection, and survival [83,84]. In retinal ganglion cells (RGCs), it has been shown that OPA1 can protect neurons from excitotoxicity [85]. OPA1 loss resulted in mitochondrial fragmentation, decreased buffering of cytosolic Ca^2+^, and sensitization of RGCs to excitotoxic injury. Similarly, the overexpression of pathogenic OPA1 mutants induced mitochondrial fragmentation and reduced motility of mitochondria in neurites of RGCs and accumulation of cytosolic Ca^2+^ in the proximity of the axonal hillock [86,87]. Additionally, Koch et al. established that MFF loss of function disturbs mitochondrial and peroxisomal dynamics, and this leads to early-onset Leigh-like basal ganglia disease [88].

In summary, mitochondrial function and dynamics are critical for maintaining neurotransmission and synaptic plasticity, with disruptions link to various neurodegenerative and neurological disorders. Understanding the mechanisms regulating the balance of fusion and fission could offer new therapeutic strategies.

## 4. Transport of Nuclear-Encoded Mitochondrial Transcripts

The mechanisms regulating the delicate equilibrium of mitochondrial fusion and fission were mostly studied at the post-translational level. However, the presence and abundance of mitochondria-shaping proteins in neurites represent another interesting level of regulation. Indeed, neurites are incredibly enriched by mRNAs encoding for mitochondrial proteins, accounting for approximately one-third of the total transcriptome. Following this, 22% of the transcriptome is composed of signal transduction-related mRNAs, while 5% are for cytoskeleton proteins and 3% are for neurodevelopment-related proteins [89]. The translation of mRNAs encoding proteins for mitochondrial ribosomes, protein import, and respiration has been measured in hippocampal neurites and retinal axon terminals [90,91], providing support for the hypothesis that local translation rejuvenates mitochondria in distal regions away from the cell body, which in turn provide the energy required for the translation of synaptic proteins [8]. Mitochondria synthesize a small fraction of their proteins from mitochondrial DNA because approximately 99% of their proteins are nuclear-encoded in mammals. These mRNAs (NEM-mRNAs) require a complex system to be transported effectively in polarised cells like neurons, which rely on the mRNA sequence together with transport granules and organelles. In these final chapters, we will review the current knowledge on the two key factors necessary to maintain the proteome of these distal proteins: the availability of mRNA and its translation. Additionally, we will discuss these mechanisms in the context of mitochondrial-shaping proteins.

### 4.1. The Sequence of mRNA

The untranslated regions (UTRs) of mRNAs are determinants of the localization: for example, longer 3′-UTRs are most likely to localize in neurites, whereas short 3′-UTRs reside in the cell body. Interestingly, transcripts with longer 3′-UTRs were found enriched in dendrites after depolarization [92]. The same mRNAs can also be differentially targeted using the 3′-UTR or the 5′-UTR (reviewed in [93]). Cis-regulatory elements (or zipcodes) are motifs found in the 3′-UTR of mRNAs, which are bound by RBPs that target mRNAs to their destination. For example, ZBP1 binds β-actin mRNA using a zip code of 54 bases at the 3′-UTR and transports β-actin from the perinuclear space to the periphery of the cell. repressing the translation [94,95]. As it was difficult to identify conserved sequences for zip codes in many studies, it has been proposed that additional mechanisms exist to regulate the localization of mRNAs. In a recent study, Loedige et al. [96] demonstrated that mRNA stability is necessary and sufficient to localize mRNAs in axons, following the principle that mRNAs with a short half-life are less likely to travel distally. The stabilization is regulated by a combination of stabilizing (5′ terminal oligopyrimidine tracts and codon optimality) and destabilizing parameters (including m6A modifications and AU-rich elements). In general, mRNAs localized in neurites have fewer destabilizing elements and relocate to the soma if they become more stable. The authors propose to use these elements as a prediction of mRNA localization in neurites, which represents a useful tool for the sake of this review. Indeed, the knowledge about the dynamics of mRNAs encoding for mitochondria-shaping proteins in dendritic spines is limited since such mRNAs have been hardly detected in axons of motoneurons because they seem to be predominantly enriched in soma and dendrites [97]. Conversely, they are highly enriched in optic nerves [90], raising the question of whether these mRNAs have different mechanisms of transport and translation among different types of neurons despite having the same sequence in all compartments. In support of this hypothesis, the abundance of these mRNA in synaptosomes changes depending on the transcript, with *Opa1*, *Mfn2*, *Fis1*, and *Dnm1l* (which encodes for DRP1) as the most enriched [98]. According to Loedige et al., the half-life of these transcripts ranges approximately from 3 to 6 h and is compatible with other mRNAs found in neurites. These transcripts have few destabilizing elements (less than 15 AREs, and *Opa1* and *Mff* also have few m6A modifications), suggesting the presence of mechanisms of transport and local translation [96]. Consistently, *Opa1* and *Dnm1l* have been found to be associated with monosomes in the neuropil of the hippocampus, similar to the translation pattern of synaptic transcripts [99]. The localization and physical properties of these mRNA are still to be properly validated with ad hoc experiments. However, the differences observed in mRNA levels prompt to suggest a complex regulation of their transport.

### 4.2. Mechanisms of Transport

Transport granules form membrane-less organelles by liquid–liquid phase separation [93], where mRNAs are bound and stabilized by RNA binding proteins (RBPs) and other components important to regulate the content and the (dys-)assembly of the granules for local translation. Interestingly, the blockage of the RNA-binding properties of FUS and TDP-43 mis-localizes them and alters the survival of neurons, suggesting that the correct localization of RBPs may depend on their binding to respective target mRNAs [100,101]. The inhibition of transcription mis-localizes TDP-43 and FUS [102,103], suggesting an additional mechanism of regulation based on the abundance of mRNA. Another mechanism seems to be related to the activity and the binding properties of VCP to RBPs in motor neurons from ALS patients [104]. ALS mutations can also shift FUS to bind mRNAs encoding mitochondrial respiratory chain components [105] or increase the binding of TDP-43 to NEM-mRNAs such as *Cox4i1* and *Atp5a1*, reducing their translation in axons [106]. Transport granules traffic using the molecular motors of microtubules, common to organelles, via direct binding to specific adaptors in anterograde direction [107,108]. However, the adaptors for dynein-mediated retrograde movement are still unknown [109]. To date, there are no known RBPs regulating the transport or the translation of mRNAs encoding for mitochondria-shaping proteins in dendritic spines. Indeed, studies aimed at investigating the RBPs bound to these mRNAs have not yet been specifically developed. An interesting candidate to regulate fusion and fission proteins in neurons could be the clustered mitochondria homolog (CLUH). CLUH binds NEM-mRNAs and components of the translation apparatus, and it prevents peripheral neuropathy [110,111,112]. Among its targets, CLUH binds Opa1, Mff, and MiD49 mRNAs in HeLa cells [111,113], regulating the translation of MFF and MiD49 in the cytosol or near mitochondria. Furthermore, CLUH recruits DRP1 to mitochondria to promote fission. The control of CLUH on these targets might be restricted to their translation because the ablation of CLUH does not impact the movement of two of its targets, *Atp5a1* and *Mdh2*, in axons. In motoneurons, CLUH is localized in cell bodies and growth cones, and it is essential for axonal growth and translation [113]. However, no data about dendrites are available due to the limited arborizations of these neurons, and further studies are required to establish whether CLUH regulates synaptic mRNAs.

**Figure 3:**
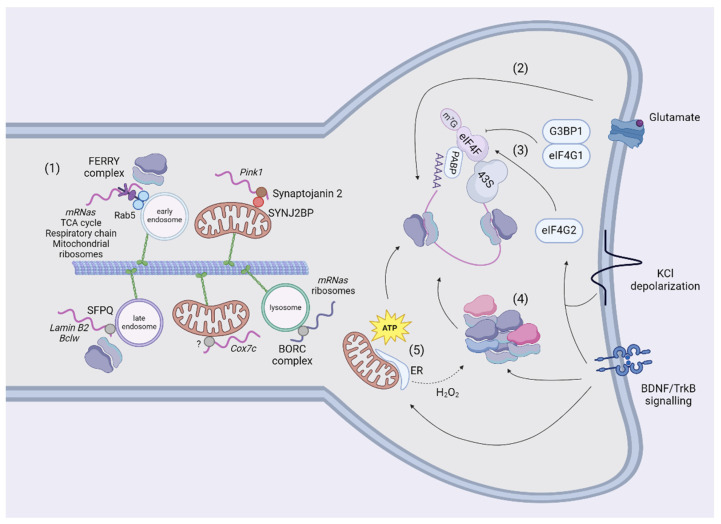
Transport and translation in neurites and spines. The mechanisms of transport of NEM-mRNAs in dendrites are still scarce (1). In neurites, NEM-mRNAs are transported using adaptors together with mitochondria (Synaptojanin 2), early (FERRY complex) and late endosomes (SFPQ). The NEM-mRNAs analyzsed in studies so far encode for proteins involved in mitochondrial metabolism, translation, and clearance. Endosomes and lysosomes can also transport ribosomes or mRNAs encoding for ribosomal subunits using the FERRY or the BORC complexes. Finally, endosomes in contact with mitochondria act as platforms to translate NEM-mRNAs. Once they reached their destination, mRNAs are translated according to the stimulus. In glutamate-dependent long-term depression, translation is resumed in polyribosomes (2). It has been shown that translating ribosomes in neurites are also organized as monosomes. G3BP1 sequesters eIF4G1 and blocks the initiation of translation in the absence of exogenous stimuli. However, BDNF or KCl-depolarization mobilizses eIF4G2 to transcripts to increase the occupancy of ribosomes (3) and induces the synthesis and assembly of ribosomal subunits (4). Mitochondria are located in proximity of to spines, where they produce ATP to sustain local synthesis (5). The tethering to the ER might potentiate translation by increasing the production of ATP. Of note, oxidative stress reshuffles the composition of ribosomal subunits (4, 5). Created using BioRender.com.

A second mechanism of transport tethers mRNAs to organelles to exploit their bi-directional and saltatory movements, which might be advantageous for responding to rapid signaling stimuli (Figure 3). Docking of organelles is an additional mechanism for regulating their movement (for a review, [114]) and might represent another type of regulation for mRNA motility to investigate in future studies. Furthermore, the tethering of mRNAs to organelles also increases the general speed of transport with respect to naked mRNAs [115,116]. Although these measurements could be distinctive and advantageous for the travel of mRNAs in general [116], there are exceptions. For example, a minor fraction of *Pink1* is not co-transported with mitochondria [115]. This slow-moving *Pink1* traffics around one-third slower than *Pink1*-tethered to mitochondria, raising the hypothesis that organelles provide support for the fast-mediated transport of mRNAs, whereas independent mRNAs move slower locally, possibly involving other unknown mechanisms. To date, mRNAs have been shown to be in traffic together with mitochondria, endosomes, and lysosomes. *Cox7a* was the first transcript described to associate and co-traffic with mitochondria in axons using the coding region [117], but this study did not focus on the linkers involved. Recently, *Pink1* has also been shown to co-traffic with mitochondria using two adaptors: the phosphatidylinositol phosphatase Synaptojanin 2 (SYNJ2) contains an RNA-binding motif and Synaptojanin 2 binding protein (SYNJ2BP) links SYNJ2 to the outer mitochondrial membrane [115]. The association of *Pink1* to mitochondria is regulated by the phosphorylation of SYNJ2 at S21 by AMPK [118]. The presence of an RBP in neurites could have different purposes than transport, as stabilizing or translating NEM-mRNAs [112] and large datasets of NEM-mRNAs co-associated and transported to mitochondria by different RBPs in neurites and synapses have not been generated. Conversely, a molecular tether and relative mRNA targets have been described for early endosomes. The FERRY complex is composed of Tbck, Ppp1r21, C12orf4, Cryzl1, and Gatd1 proteins, and it tethers NEM-mRNAs to early endosomes through interaction with Rab5 [119]. This complex binds mRNAs encoding components of the mitochondrial matrix, mitochondrial ribosome, cellular respiration, and tricarboxylic acid cycle in HeLa cells. Some of its targets (*mdh2* and *uchl1*) have been validated by FISH to be localized to early endosomes in cell bodies and neurites. In these experiments, the FERRY complex seems to reside in spines [119]. However, the FERRY complex does not co-precipitate with mRNAs encoding for mitochondria-shaping proteins, but the knocking out of single FERRY subunits reduces the levels of these mRNAs, potentially suggesting an indirect mechanism of regulation of their abundance. Finally, Splicing Factor Proline and glutamine-rich (SFPQ) assembles and traffics *Lmnb2* and *Bcl2l2* in RNA granules in axons, which colocalize with ribosomes in the proximity of mitochondria [120]. SFPQ co-immunoprecipitates with Rab7 and is localized to Rab7a-positive endosomes in axons [116]. An interesting candidate to transport NEM-mRNAs is the BLOC-one-related complex (BORC), which tethers mRNAs to lysosomes in axons [121]. The authors investigated solely the trafficking of Rps7 and Rps27A mRNAs, despite BORC-KO axons being depleted of mRNAs encoding both ribosomal and OXPHOS proteins. The absence of the BORC complex also reduces the translation of these mRNAs, but it is unclear whether this effect is directly mediated by BORC’s transport of NEM-mRNAs or is secondary to the lack of ribosomes.

Collectively, these studies provide a glimpse into the complex regulation of NEM-mRNAs in neurites and highlight the potential for investigating other mechanisms related to rapid adaptation to external stimuli, which is crucial in synapses. It is also worth noting the limitations of these studies, as the anatomical structure of certain neurons might reveal regulators that are more prominent in axons than in dendrites. Therefore, these regulators should be specifically tested in synapses, and studies focused on identifying novel regulators should be developed specifically on dendrites.

## 5. Translation of Nuclear-Encoded Mitochondrial Transcripts

Growth cones, which are the tips of axons devoted to their growth, sense extracellular cues to direct the extension of axons during development. The presence of a local, autonomous translation in neuronal compartments has been shown in experiments where severed neurites attached to the plate stopped responding to chemotropic cues in the presence of protein synthesis inhibitors [122]. This so-called local protein synthesis (LPS) in synapses has been induced in primary neurons using poly-D-lysine coated beads, which trigger the clustering of presynaptic proteins and the formation of pre-synapses. Specifically, these studies showed the local translation of Snap25 and β-catenin [123,124]. Translation in dendrites occurs at 50% near synapses and it is correlated with spontaneous Ca^2+^ activity following spine stimulation by glutamate uncaging [125]. The majority of studies focused on translation initiation because some of the first evidence regarded the recruitment of initiation factors and activation of LPS in spines in the presence of BDNF for long-term potentiation [126,127]. The initiation of translation is regulated by the formation of the 43S pre-initiation complex, which is recruited by the eIF4F complex to form the initiation complex that scans the 5′-UTR until the starting codon (for a review, [128]). The eIF4F complex is composed of the cap-binding protein eIF4E, the RNA helicase eIF4A and the scaffolding protein eIF4G. In the absence of exogenous stimuli, G3BP1 interacts with eIF4E and inhibits translation, regulating the genesis of mushroom spines [129]. Upon depolarization, eIF4G2 has been found to bind the 5′-UTR of dendritic transcripts, increasing the ribosome occupancy in uORFs and CDS translation [92] (Figure 3). Of note, *Timm23* (the core component of the TIM23 complex that imports proteins across the inner mitochondrial membrane [130]) and *Nsun3* (a matrix-localized methyltransferase regulating mitochondrial translation [131]) were more translated in stimulated dendrites, suggesting an effect to potentiate the import of newly synthesized proteins to mitochondria. Another study showed that translation may also be resumed on polyribosomes, which are stalled in unstimulated spines, for metabotropic glutamate receptor long-term depression independently of translation initiation [132]. Authors propose that this regulation is more rapid and transcriptionally effective in responding to stimuli with respect to translation initiation. Data about the protein content in spines are scarce and conflicting, probably due to the limited amount of these proteins. Indeed, several mitochondria-shaping proteins have not been measured in screenings from synaptosomes or were detected only in synaptosomes from specific neuronal populations [133,134]. Interestingly, Jung et al. combined axon-TRAP with metabolic labeling of RNAs to measure the translatome in axons of RGCs [90]. Authors report that mRNAs encoding for mitochondria-shaping proteins in axons are differentially translated in axons independently from having a pro-fusion/fission function. Another recent observation has been reported in dendritic spines [92]. In this study, authors mapped the translatome using a construct expressing TurboID tagged with the post-synaptic protein PSD95, showing that NEM-mRNAs are translationally silent in resting conditions, and the translation initiation factor eIF4G2 is required to translate dendritic NEM-mRNAs upon depolarization. However, the translation of *Opa1*, *Mfn1/2*, and *Dnm1l* was not altered. It is possible that other stimuli control the abundance of these proteins, as has been shown for *Pink1*, which is associated with mitochondria after insulin administration [118].

The organization of ribosomes in spines is another mechanism to regulate translation (Figure 3). Indeed, ribosome dynamics and activity are subjected to rapid regulation since assembly and translation of ribosomal subunits occur in seconds after BDNF administration [135]. In isolated dendrites, ribosomal RNAs associate with existing ribosomes to promote ribogenesis and potentially potentiate the synthesis of dendritic proteins [136]. The authors also reported that the addition of H_2_O_2_ reduced the synthesis of new ribosomal subunits and increased the incorporation of a small subset of ribosomal proteins. This observation suggests that the physiological state of dendrites modulates the composition of ribosomes and that mitochondrial dysfunction (namely oxidative stress) could probably signal a reshuffling of ribosomal subunits. The hypothesis from these data has not been investigated in this work, but it will be interesting to measure if and how the local translatome changes according to different mitochondrial dysfunctions and possibly the composition of ribosomes. Ribosomes appeared to be organized in polysomes in electron micrographs of stimulated spines [137,138]. However, an interesting study highlighted that ribosomes are differently organized in neurons, with a prevalence of polysomes in cell bodies and monosomes in neurites [139]. Monosomes—previously considered translationally inactive—probably couple the limited space available in spines to the necessity of translating a plethora of proteins, including preferentially synaptic proteins but also NEM-proteins. As we mentioned in the previous paragraph, NEM-mRNAs are transported together with organelles [116]. Additionally, 70% of the proteins interacting with the FERRY complex are ribosomal subunits, suggesting that FERRY could directly link entire ribosomes to early endosomes [119]. Since the FERRY complex is associated with ribosomes, it might have an indirect but local effect on the translation of mRNAs related to mitochondria-shaping proteins, which are reduced in the absence of the knocking out of single FERRY subunits. Additionally, ribosomes also co-traffic with these mRNAs, forming supercomplexes on organelles devoted to translation platforms to supply new proteins to axonal mitochondria. In developing axons of RGCs, local translation of *lmnb2* was measured on Rab7a-positive endosomes paused in contact with mitochondria [116].

Several lines of evidence suggest that mitochondria are heterogeneous in neurons. In primary neurons, mitochondria decrease their membrane potential and increase reactive oxygen species production with the distance to the soma [140]. Interestingly, the same distal mitochondria imported a mitochondrial reporter slower than perinuclear mitochondria. Moreover, synaptic mitochondria have a different Ca^2+^ buffering capacity and membrane potential than non-synaptic mitochondria extracted from mouse brains [19,141], and the bioenergetic properties of these mitochondria are prone to decline with age [19,141,142]. A common shared hypothesis is that mitochondria accumulate damage when they traffic to reach distal areas, a process that in humans can last even days [143]. Mitochondria are normally positioned at LPS sites in different neuronal compartments, where they support protein synthesis and generate ATP (Figure 3). For instance, the respiration of stalled mitochondria along the axons induces the maturation of filopodia into branches by generating sites of LPS [144], and the reduction of the mitochondrial mass by PGC1α inhibits spinogenesis and synaptogenesis [145]. In dendritic spines, the inhibition of mitochondrial function using antimycin or oligomycin decreased newly synthesized proteins [8]. This effect was also observed in single-stimulated spines depleted from functional mitochondria, suggesting that mitochondria are strategically positioned close to spines to sustain LPS during plasticity. In the same study, MFF induced fragmentation of mitochondria in spines and reduced protein synthesis in stimulated spines, pointing to the importance of the morphology of mitochondria for effective translation. LPS also fosters inter-organelle communication. Following axotomy, the glucose-regulated protein 75 (Grp75) was translated at the site of injury, which increased the tethering of ER and mitochondria, increasing the levels of ATP. Hence, the generation of ATP is controlled locally and promotes the regeneration of severed axons [146]. Interestingly, the ER is also found to be localized near mitochondria at sites of branching [144], and the tethering of these organelles could be mediated by the LPS of Mfn2.

In conclusion, LPS in spines is essential for neuronal function, with mitochondria playing a critical role by providing energy and facilitating synaptic plasticity. The translation of mitochondria-shaping proteins may be fundamental for maintaining mitochondrial structure and function, thereby directly influencing LPS. Elucidating the mechanisms by which stimuli and mitochondrial dynamics affect the local synthesis of these proteins is crucial for gaining insights into neuronal adaptation and health.

## 6. Conclusions

The regulation of mRNA transport and local translation within neurons is essential for maintaining the proteome of distal mitochondria, which is crucial for energy production and synaptic function. A significant portion of the axonal transcriptome is dedicated to mRNAs for mitochondrial proteins, highlighting the importance of local translation in sustaining mitochondrial function in regions far from the cell body. This process relies on transport mechanisms involving RBPs and organelles like mitochondria, endosomes, and lysosomes, which ensure the precise localization and translation of these mRNAs. Factors such as untranslated regions (UTRs), cis-regulatory elements, and mRNA stability play key roles in this regulation.

In dendrites, local translation and the regulation of mRNAs encoding mitochondrial-shaping proteins could be crucial for synaptic plasticity and neuronal health. The dynamics of these mRNAs, including their transport and local translation, may influence the morphology and function of mitochondria, thereby affecting the overall energy status and responsiveness of synapses. However, the knowledge about the specific mechanisms regulating these transcripts in dendrites remains limited. The presence of mitochondria-shaping protein mRNAs in synaptosomes suggests a local regulation. For instance, extracellular cues, which trigger local translation initiation and ribosome reorganization, could induce the rapid synthesis of these proteins at synapses. However, data on the dynamics and local translation of mRNAs for mitochondrial-shaping proteins in dendrites are scarce, and further research is necessary to fully elucidate the stimuli and pathways regulating the translation of these proteins. Understanding these regulatory processes is essential for comprehending how neurons maintain their functionality and adaptability, particularly under conditions of stress or injury. Such insights may elucidate novel therapeutic targets for neurological disorders in which mitochondrial dysfunction and impaired synaptic plasticity play a central role.

## Data Availability

No new data were created or analyzed in this study. Data sharing is not applicable to this article.
